# Beyond the neoliberal label: A historical perspective on sexual actors and responsibility in HIV prevention in England (1986–2023)

**DOI:** 10.1177/13634593241238862

**Published:** 2024-03-18

**Authors:** Alvaro Martinez-Lacabe

**Affiliations:** University of Sussex, UK

**Keywords:** ethnography, HIV/AIDS, research methodology, theory

## Abstract

Framed across three distinct periods of the history of neoliberalism and the HIV epidemic in England, this article conducts a detailed examination of the concept of personal responsibility and its contested uses within HIV prevention. The article questions the theoretical potential of neoliberal subjectivities to comprehend behaviours related to the pharmaceuticalised governance (or lack thereof) of gay men’s sexual health, exploring the gap between theories emphasising individual responsibility and the practical experiences of gay men. The analysis draws on testimonials from gay men in oral history interviews and archival sources. The article illustrates how the pervasive notion of personal responsibility in England has been co-opted by neoliberal ideologies, leading to the stigmatisation of gay men whose sexual behaviours diverge from public health mandates. The widespread stigmatisation resulting from this ideology underscores a significant limitation in the theoretical framework of neoliberal subjectivities. This constraint extends beyond merely failing to grasp the complexity of sexual behaviours; it also reflects a lack of understanding of any other behaviour related to public health. Therefore, the article concludes by advocating the necessity of employing and constructing alternative theoretical frameworks to comprehend the pharmaceutical governance or lack thereof of gay men’s sexual health. Through a concise autoethnography of the authors’ pharmaceutical sexual health governance, the article introduces the concept of biocommesuration as an illustrative analysis that transcends the limitations of neoliberal subjectivities.

## Introduction

Following Venugopal’s (2015) critical examination of the term neoliberalism, Bell and Green (2016) cautioned about the need to reconsider the value of neoliberal critique within the intersections of public health and social sciences. This neoliberal critique, often intertwined with analyses of (bio)governmentalities (Keogh, 2008), and (bio)sexual citizenship (Hakim et al., 2022; Young et al., 2020), gave rise to different health-related subjectivities embodied by metaphorical *neoliberal actors*. These subjectivities typify actors who embody values associated with neoliberal ideology, such as personal responsibility, market choice, and rational risk analysis. These neoliberal subjectivities have been employed to analyse a variety of health-related behaviours in different socio and geographical contexts, including vaccination hesitancy (Sanders and Burnett, 2019), parenting/mothering and vaccination (Reich, 2016), indigenous oral health (Poirier et al., 2022), financial debt and mental health (Sweet, 2018) or pharmacopornographic subjectivities (Preciado, 2013), among others. Some of these studies and critiques emphasised particularly personal responsibility to investigate health behaviours, yielding mixed results that, at times, led to the stigmatisation of the subjects under study. Notably, studies incorporating additional factors such as institutional racism or psychosocial elements provided more robust analysis and less stigmatising outcomes compared to those solely focussing on the notion of neoliberal responsibility, choice rhetoric or risk analysis. The same could be said of studies on HIV prevention using this type of neoliberal critique. The idea of *the neoliberal sexual actor* emerged promptly, being one of the precursors of these neoliberal health-related subjectivities that continue, in varying degrees, to influence research on HIV prevention (Adam, 2005; Adam and Rangel, 2014) and PrEP (Gaspar et al., 2022; Preciado, 2015; Sandset, 2019; Sandset et al., 2023; Thomann, 2018). This is not to disregard the impact that neoliberal policies and neoliberal (bio)governmentalities have on the lives of those affected by healthcare cuts, the co-option of PrEP activists’ messages and success by politicians, or as some of the above-mentioned articles explain, the role of neoliberal ideology in building structural and psychological barriers to health.

Using historical analysis, this article aims to explain how healthcare for gay men could be strengthened by moving beyond counterproductive and facile applications of neo-liberalism, especially the personal responsibility rhetoric. In this sense, I argue that neoliberal (bio)governmentalities might be understood from resistance positions, ranging from practices linked to the care of the other, solidarity and the knitting of the community (Lazarus et al., 2021; Martinez-Lacabe, 2019) to PrEP commodity activism (Martinez-Lacabe, 2021), a type of activism that navigates neoliberal structures to provide access to PrEP for those who need it when governments do not fulfil their due care. Further, this article advocates for the development of new theoretical frameworks to understand how gay men (and others) elaborate their decision-making processes. Finally, this article introduces biocommensuration (Ecks, 2022) as a theoretical framework to illustrate the process of valuing the use of pharmaceuticals encompassing aspects usually disregarded by neoliberal subjectivities, such as intimacy, pleasure, phallocratic and ageist pressures, etc. Biocommensuration is a sophisticated valuation process that extends beyond economic measures, incorporating criteria such as pleasure, intimacy, and health. It provides a nuanced understanding of decision-making, transcending monetary considerations for a holistic perspective.

Mainly framed in three historical periods of the epidemic—before the introduction of highly active antiretroviral therapy (HAART), during HAART and before the PrEP era, and during the PrEP era—I begin by examining contesting forms of individual responsibility and the construction of the ‘responsible gay’ in England by both proponents of neoliberal policies and AIDS activists. In addition, testimonies provided by participants who focussed on responsibility will be analysed to elucidate the adequacy of constructing PrEP users in the UK as neoliberal sexual actors. In doing so, the article aims to determine the extent to which the concepts of personal responsibility and neoliberal sexual actors enhance gay men’s sexual health or contribute to obscuring meaningful aspects in gay men’s decision-making processes.

## Methods

This article utilises testimonies from the National Lesbian and Gay Survey (NLGS). The NLGS spans a period of 28 years, from 1986 to 2004, with a total of 724 participants who answered questions about varied subjects, including opinions about government attitudes towards AIDS research and subjective experiences with AIDS and AIDS activism. These testimonies were used to examine perceptions of personal responsibility and how to link these to the idea of a neoliberal sexual actor. In addition, this project draws on testimonies from oral history interviews with first-wave PrEP users (*n* = 15). Recruitment of interviewees was made online through different channels. All participants were provided with a consent form with information about the nature of the project and their right to withdraw from it at any time. Finally, the article includes an autoethnography that analyses the author’s valuation process concerning forms of pharmaceuticalised governance of HIV prevention and personal responsibility.

## Responsible gays and personal responsibility and the rise of neoliberalism

Influential discourse on personal responsibility can be found in Thatcher’s (1949) speeches dating back to the late 1940s (Dartford Chronicle, 19 August 1949). Her references and calls to ‘personal responsibility’ were a constant throughout her political career and, along with the ideas of ‘freedom’ and ‘less government intervention’, was one of the ideological axes on which her political practice revolved. This conception of the individual as the core of various governmental matters never vanished; on the contrary, it remains one of the strategies of contemporary neoliberal governments. In July 2015, Health Secretary Jeremy Hunt delivered a speech entitled ‘Personal Responsibility’, claiming that ‘to deliver the highest standards of health and care the people who use those services need to play their part too: personal responsibility needs to sit squarely alongside system accountability’ (Hunt, 2015). On March 2022, Hunt’s successor Sajid Javid stated: ‘I firmly believe in individual responsibility, which is why we need to do so much more on prevention and personalisation’ (Javid, 2022). In short, neoliberal programmes, including New Labour’s (Hall, 2011; Halpern et al., 2004), aim permanently to re-signify the fragile balance between personal responsibility and the welfare state, to remind citizens of their duty in safeguarding a national health system that is in constant threat of economic collapse. As implied, the trope of personal responsibility is not new.

What is *neo*, is the active attempts to involve and link personal behaviours and health outcomes with the economic sustainability of health systems, favouring the increasing privatisation of said systems and the reconfiguration of patients as health consumers. Inspired by the American model, the British neoliberal health system claims that health consumers are responsible for making the correct choices while dismissing structural and racial barriers to health care services. For example, the discussion of the Black Report (1980) commissioned by the Labour Party provided strong evidence of class affecting life expectancy regardless of lifestyle factors. Disregarding the evidence, the conservative party did not support the proposals aimed at addressing class barriers and reducing poverty. On the contrary, Edwina Currie, minister of health from 1988 to 1990, distributed a paper among their fellows highlighting the importance of ‘conservative philosophies of personal responsibility’ (Snelling, 2014: 36). This rhetoric has targeted gay men for decades in England and plays a key role in the construction of the res/irresponsible homosexual.

The construction of the *responsible homosexual* versus the *irresponsible homosexual* occurred on two different levels and was shaped by two overlapping historical events. The first, undeniably, was the AIDS epidemic; the second was the promulgation of Section 28 of the Local Government Act (1998). Section 28 grew out of the fear and hatred spread by the panic around AIDS and the general obsession with the return to *family values* under Thatcher—in turn linked to the development of neoliberal policies as well as the tensions between individuals and families, on the one hand, and wider society, on the other. During parliamentary debates surrounding the passing of the law, there were constant references to what Smith (1992) critically defined as the *responsible homosexual*, someone who was assimilated by the State and posed no threat to the moral order. Further developing this idea, Smith stated:

The responsible homosexual is more than just closeted and does not just seek acceptance. He or she attempts to achieve acceptance in the terms promised by official discourse and by furthering the demonization and exclusion of dangerous gayness. The responsible homosexual therefore functions as the ‘contra’ force within the community; his or her presence allows right-wing politicians to speak in our name as the representatives of the true homosexuals. Queer activism therefore subverts the differentiating logic of official discourse by inviting all lesbians and gays to identify with the dangerous gayness position. (Smith, 1992: 206)

By drawing an analogy between the figure of the ‘contra’ against the Sandinista revolution and the responsible homosexual, Smith warned about the dangers that the latter posed to queer activism. At the time Smith was writing, queer activism was largely focussed on AIDS treatment and prevention, and British ACT UP, like its counterparts in the States, was one of the groups that was more vocal about the health crisis. Activists themselves thought about the need to be responsible towards others during the AIDS epidemic. This is the second level at which the *responsible gay* was constructed in England during the 1980s, this time linked to safe-sex practices. The role of individual responsibility and its moral weight, in this case, linked to the survival of the collective, is present in the following quote from Peter Tatchell:

Choosing safe sex is thus not only a matter of individual survival, but for male homosexuals it is also a question of the collective survival of the lesbian and gay community and its achievements. Already we have lost some of the finest people in the movement – political activists, writers, volunteer workers and just plain nobodies who nevertheless made their own unsung contribution to gay freedom by living their lives with pride and dignity. Most of these people contracted the virus long before the dangers were fully understood and publicised. They share no blame. But it is a different story for others who today continue to take risks despite the known dangers. For in this AIDS-threatened era, playing dangerously – the refusal to take care of oneself or others – is a new form of self-oppression. (Tatchell, 1986: 41)

Tatchell’s words echo(ed) the thoughts of many regarding HIV prevention: self-care is key to preserving/taking care of the community. This argument remains relevant in HIV prevention, and it is echoed by many PrEP users in different studies (Martinez-Lacabe, 2019; Rosengarten and Murphy, 2020). At that moment in history, when gay identity and community survival were strongly linked to safe sex, Tatchell’s words contributed to deepening the division between the *responsible* and the *irresponsible* gay, without acknowledging the role of psychological and socio-structural factors in the transmission of HIV, similar to the way neoliberal policymaking does not recognise such factors. Allegedly, in a context where there was no effective treatment for HIV, it seemed appropriate that health policies and discourses focussed on the rights and wrongs of individuals (Crawford, 1994; Dodds, 2002). Despite all this, labelling Tatchell’s words as neoliberal would be to dismiss his anxiety at the loss of collective action and common responsibility, both dimensions deeply related to the construction of the Keynesian welfare state. Finally, a key concern might arise from the seemingly contradictory fact that alongside the responsible, *neoliberalised* self, the response to HIV, especially in the early days, had a pronounced community and collective character (Berridge, 1996; Watney, 2000; Weeks et al., 1996). This alleged paradox stems from ‘the radical academic discourse of “neoliberalism”’ that ‘frames the relationship between collective action and individualism simplistically as opposition between the good and the bad’ (Barnett, 2005: 11), which often results in moralistic labelling. Polarised dichotomies like this prevent the reconciliation of individual responses and/or personal responsibility with collective actions. Collective actions in the realm of health practices cannot be separated from individual actions: the action of using condoms or PrEP requires individual manoeuvres that have potential positive outcomes in terms of public health. Furthermore, personal responsibility-related subjectivities in this era were weaponised by members of the conservative party as well as HIV activists with opposite conceptualisations of responsibility.

## Personal responsibility and community before HAART: ‘Those we love’

The NLGS is an important source for the testimonies and opinions of gay and lesbian people on topics affecting them. The issue of personal responsibility emerged as a central theme in many of the participants’ responses within the AIDS directive, and it was widely understood as a necessary tool to counter the epidemic within the framework of an instrumentalised gay community. The following testimony from a 34-year-old man living in Huddersfield in 1986 is a response to the questions described in the Methods Section. After explaining that he was unadventurous in terms of sexual practices, the respondent focussed on the issue of responsibility. The testimony reflects the pervasive polarisation of the question of the balance between state accountability and personal responsibility:

I suppose for myself, I am a conservative on such matters, but that doesn’t mean I assume that others will want that for themselves, however, I cannot agree with the pseudo-Marxist ‘AIDS is all about society and there is no role for personal responsibility’. As a liberal, I believe gay liberation is about individual self-awareness and independent thought and action is most fundamental, and that must include taking ultimate responsibility for ourselves and those we love. (NLGS respondent 367)

This testimony is consistent with Thatcher’s critical words towards those who demanded more state accountability. However, is this testimony a neoliberal approach to HIV prevention? It seems that the critique of the ‘pseudo-Marxist’ approach is less an embrace of neoliberalism than a plea for a more nuanced position that acknowledges a dialectic between the individual and society. Developing a more nuanced position in the debate demands an acknowledgement that HIV education by activists and AIDS organisations in the 1980s was strongly shaped by the concept of individual responsibility, and it was not until the 1990s that material on HIV education started talking about shared or common responsibilities (Dodds, 2002).

Along with these complex understandings of personal responsibility and the difficult relationship between the Thatcherite government and AIDS activists, it is necessary to acknowledge the link between the care of the individual (*care of the self*) and the care of the community, the care of *those we love*. The care of *those we love* must be contextualised at a time when acquiring HIV was usually linked to traumatic losses. In that setting, strategies that involved individual responsibility were deemed imperative for the survival of the gay community. However, the notion of the gay community and its link with responsible behaviours remains an ambiguous one and is perceived in different ways by respondents in the NLGS:

As for the gay community itself, well, the real problem is that it is not a community and the organised part of it is merely the tip of an enormous, concealed iceberg. Nevertheless, all the signs are that gay men have reacted to AIDS fairly responsible [sic], though without any real encouragement from society in general. There are limits to what we can do, and in any case, many of us will always feel that to renounce a full expression of our love is too high a price to pay for safety. I am not sure that this is so wrong. Death after all stalks us at every turn, though modern societies do their damnedest to forget that fact. (NLGS respondent 183)

There is a clear recognition of the role of gay men in acting responsibly in the face of the epidemic, just as there is an acknowledgement of the difficulties that acting responsibly entails. Most importantly, this testimony calls into question the idea of the gay community, understood as a section of the population characterised simply by being gay, and a feeling of fraternity and mutual care. His reference to the ‘organised part’ of the community has to do with the concept of *community* used in the promotion of sexual health in England, which has often been a controversial issue among some members of the gay population (Russell, 2005). On the other hand, overly individualistic approaches have often resulted in victim-blaming (Dodds, 2002) instead of collective empowerment. In fact, the 1980s idea of the *irresponsible gay* versus the *responsible gay* permeated the mentality of many, as illustrated by the following participants in the NLGS:

I think the responsible gay community are well educated but we have our fair share of irresponsible members. (NLGS respondent 168)

In so far as there is truly a gay community it has done a great deal to educate itself, but a lot of gays do not belong to that community (and many of those who do persist in running grave risks for themselves and others). There should be much more publicity (e.g., in public toilets). (NLGS respondent 282)

The two testimonies above reveal a clear distinction between the responsible gay, men who have made a great effort to educate themselves, and those who ‘persist in running grave risks for themselves and others’.

In summary, until the early 1990s, when HIV began to be understood as a manageable chronic condition, prevention interventions were mostly shaped by individualistic approaches that triggered the polarisation of the debate on personal responsibility. The former included multiple characterisations and understandings, sometimes in opposition to one another, of the gay community. Before the appearance of effective antiretroviral treatment, the morals of personal responsibility in England were concerned with blaming those who would not engage in safe sex, contributing to creating a divide between HIV-positive gay men and HIV-negative gay men. However, in the reasoning of those who blamed men not engaging in safe practices, there were no allusions to the financial consequences that such practices might carry for the allocation of HIV resources but rather to the survival of the gay community.

## Personal responsibility and *neoliberal sexual actors* after HAART

For some time within the study of HIV prevention, the concept of the *neoliberal sexual actor* has been deployed as an analytical tool to interpret sexual behaviour(s) in economic terms (Adam, 2005, 2006; Adam and Rangel, 2014; Sandset, 2019; Thomann, 2018). This model, emphasising neoliberal responsibilisation, is linked to the vision of those American neoliberals who began applying a market analytical framework ‘to reveal in non-economic processes, relations, and behaviour a number of intelligible relations which otherwise would not have appeared as such—a sort of economic analysis of the non-economic’ (Foucault, 2008: 243). With a focus on responsibility, this section aims to reflect on the history and uses of this model of sexual subjectivity in HIV prevention literature and to answer what is distinctive about neoliberal responsibilisation within HIV prevention.

The term *neoliberal sexual actor* was first deployed in a qualitative study based in Toronto that examined barebackers’ discourses (Adam, 2005). The article, written 10 years into the HAART era and before *The Swiss Statement* as the first articulation of Treatment as Prevention (Vernazza et al., 2008), argues that despite barebacking being described as pathological and irrational behaviour, ‘barebacking discourse displays a remarkable consistency with the leading contemporary strands of moral reasoning circulating in advanced industrial societies today’ (Adam, 2005: 333). In other words, barebackers were not pathological subjects but individuals who fit within the grid of the *neoliberal sexual subject* because of their reproduction of neoliberal rhetoric. In a historical overview of the neoliberal sexual subject, Adam explains that AIDS services organisations (ASO) acted as agents of responsibilisation by ‘calling up gay men to re-make their sexuality’ whilst homosexuality ‘was to be refashioned into a model for good citizenship: tamed, responsible, and governed by the safe sex ethic’ (Adam, 2005: 334). As part of the construction of the neoliberal sexual subject, the question of responsibility features a prominent role, and it is always understood as individual, not collective responsibility.

Drawing on discourse analysis, the article characterised some HIV-positive barebackers as neoliberal sexual subjects who, mimicking the language of ASOs, recurred to responsibilisation to justify potential HIV transmission. The following excerpt introduces the notion of the sexual neoliberal subject, represented as a human calculator in a commodified landscape of sexual opportunities and HIV prevention options:

The neoliberal view constructs human actors as rational, adult, contract-making individuals in a free market of options. It does not account for the much more complex motivators and vulnerabilities that characterize real human interaction and it denies the vulnerabilities, emotions, and tough dilemmas faced by people in their everyday lives. In terms of this study, the rationale advanced for unprotected sex by barebackers denies such circumstances and dilemmas that go into unprotected sex as a partner’s erectile difficulties, momentary lapses and trade-offs, personal turmoil and depression, disclosure and intuiting safety, and indeed love. (Adam, 2006: 344)

The limitations of constructing human actors *as ‘rational, adult, contract-making individuals in a free market of options neoliberal view’*, are clearly and critically exposed. However, drawing on the moral reasoning produced in the interviews, real HIV-positive barebackers are paradoxically characterised as neoliberal sexual actors. This characterisation does not acknowledge the barebackers’ own vulnerabilities, emotions and tough dilemmas in their everyday lives. As warned, the use of the concept of the neoliberal sexual subject risks resulting in moral criticism of the study participants: ‘Interviews with those men who have abandoned safer sex practice show just how attuned their moral reasoning is with this neoliberal model of human subjectivity’ (Adam, 2005: 344). But as Adam acknowledged, ‘even among barebackers who invoke neoliberal discourse most in the care of the self, there are clearly a host of competing discourses, drawn from romance, masculine adventure, gay solidarity, communitarianism, and so on, that can come to the fore, according to circumstance’ (Adam, 2005). In contradiction with the construction of barebackers as neoliberal sexual actors, the previous statement already hints at the necessity of moving beyond this model of embodied subjectivity to analyse sexual behaviours.

However, a decade later, the model of *homo oeconomicus* was deployed in the same fashion to examine ‘the presence of neoliberal ideology in the narratives and subjectivities as it relates to HIV prevention in the lives of young gay and bisexual men living in New York City’ (Siconolfi et al., 2015: 554). Acknowledging similar limitations, the article concluded that despite the presence of neoliberal themes in participants’ conversations such as responsibility, entrepreneurship, and citizenship, the *homo oeconomicus* metaphor failed to address structural factors that shaped the sexual experiences of these young men. This acknowledgement reinforces the idea that labelling sexual subjectivities as neoliberal is not helpful for the analysis of sexual behaviours. The presence of neoliberal ideology in the subjects of the study is a reminder of how the language often used in neoliberal programmes has permeated everyday language and practices (Turken et al., 2015), without this meaning that participants embrace such an agenda. The fact that, as part of the recruitment process, researchers handed *business cards* to young gay and bisexual men, along with a monetary incentive, illustrates how business language and practices have permeated social research. However, labelling said research as neoliberal because the use and rhetoric of market language would require further justification.

Thomann’s (2018) and Sandset’s (2019) articles offer a less stigmatising application of the concept of the neoliberal sexual actor. The concept is used to analyse HIV prevention campaigns and not actual sexual behaviours, or decision-making processes. Thomann (2018) rightly points out how strategies to biomedicalise and pharmaceuticalise HIV prevention risk undermining investment in community responses. In addition, he warns that this focus on biomedical and pharmaceutical responses tends to divert attention from the *messy* aspects of the epidemic, such as racism and inequality. Despite this fundamental warning, the article bolsters Adams’ *neoliberal sexual actor* by adding the parameters of pre-emption and pharmaceuticalisation. Throughout the article, there is an unequivocal correlation between personal responsibility and neoliberalism as can be deduced from the indistinct use of the ‘responsible sexual actor’ and the ‘neoliberal sexual actor’. This manifests the problematic relationship between the concepts of personal responsibility and neoliberalism. At this point, it is convenient to remember that in the early twentieth century, medical institutions in Britain had already begun to develop a discourse on personal responsibility, often related to hygiene. Health promotion campaigns such as *Health Week(s)* in London started in 1912 with the goal ‘to arouse a sense of personal responsibility for keeping well’ (Churchill, 1924: 18). Therefore, the link between personal responsibility and preventive medicine was established before those who call themselves neoliberals came onto the scene.

Sandset’s (2019) article rightly stresses the fact that health campaigns based on neoliberal concepts of personal responsibility run the risk of leaving certain populations behind. Analytically speaking, Sandset’s thorough clarification of the concept of the *neoliberal sexual actor* (2019), reiterated that ‘neoliberalism is about the application of the economic grid to social phenomena’ (Foucault, 2008: 239); There was no mention, however, of the Foucauldian question ‘to what extent it is legitimate and to what extent it is fruitful, to apply the grid, the scheme and the model of homo oeconomicus not for all economic actors, but for all social actors in general?’ (Foucault, 2008: 268). I argue that the latter question should be of greater interest and relevance to those working at the intersections of sexual health and the humanities and social sciences due to the ethical dimensions involving the stigmatisation and labelling of individuals or certain populations, as explained below.

The application of this type of analysis, drawing on a recurring metaphor from the history of economic ideas, presents multiple limitations, including the tendency to stigmatise legitimate concerns and individual actions about one’s health care by labelling them as ‘neoliberal’. For example, Briggs and Hallin (2007) characterised the neoliberal subject, as ‘the patient-consumer who actively and responsibly seeks health information and produces health by regulating his or her choices accordingly’ (p. 44). Sandset refers to the incorporation of desire and pleasure as key components in a ‘sort of neo-liberal investment into the personal health of the individual’ (Sandset, 2019: 667), which might suggest that the relation between pleasure, desire and health is as a neoliberal phenomenon when it is a long-standing practice that precedes capitalist societies. In relation to the problematic simplification of free individuals and choices in supposedly neoliberal scenarios, Nikolas Rose argued that:

You never have the simple scene of neoliberalism; it’s about a complex relationship between the choices of free individuals and the attempt to shape those choices for the good of society as a whole. This is why I call them advanced liberal societies rather than neoliberalisms. Neoliberalism is a program, but it is not the way things are governed. (Avellaneda and Vega, 2012: 11)

This distinction of neoliberalism as a programme and not as the way things are governed clashes with the multiple interpretations of neoliberalism as governmentality. Rose explains that the neoliberal governmentality project, in which everything is reduced to the government of homo oeconomicus, cannot work since aspects such as health and border management require collective government (Avellaneda and Vega, 2012). Rose clarifies that the so-called neoliberal programmes of the 80s and 90s were highly focussed on communitarianism or the government of the population through communities (Avellaneda and Vega, 2012). In fact, the public health proposals that refer to the health of gay men and MSM are an example of this management of health through the community. Beyond the neoliberal rhetoric of responsibility, active citizenship and risk, these proposals were and are often based on caring for communities. This fits better with a way of governing that would not be called neoliberal but an advanced, liberal and democratic way. Of course, there are also limitations to this interpretation where aspects such as classism, racism or religion would have a determining role in the ability to make decisions about one’s own health. The advantage, however, is that moralistic concepts such as personal responsibility lose the extreme relevance that is attributed to them.

In fact, contrary to framing PrEP users as neoliberal actors, some participants saw PrEP as a catalyst for activism, resistance, and social change (Martinez-Lacabe, 2019). While all participants in this study embraced the notion that ‘the responsibility for preventing HIV infection is a question of protecting oneself’ (Adam, 2005: 198), most also viewed PrEP as a means to care for both themselves and their community, challenging the notion of health solely as an individual concern. The study uncovered diverse perspectives and tensions regarding choice and responsibility among gay men. For instance, a transgender gay participant noted that preserving the health of other gay men through antiretrovirals could be considered a byproduct of PrEP:

Ultimately, it is about me because it has to be [. . .] I can only be responsible for me, I can’t be responsible for somebody else. However, what I can do is by getting tested and the rest of it, then I can make sure that if they do have something, they’re getting treated and therefore yes, I wouldn’t pass it on before I got my test results, but at least I got tested and I made it in the shortest amount of time that I could, of potentially passing something on. Yes, it is looking after your partners as well, but at the end of the day, you can only really be responsible for yourself. (Daniel. Age 50. Lichfield)

Here is an explicit recognition of responsibility as something that cannot be transferred. This way of thinking is based on the recognition that other people have a sovereign right to decide on their bodies, and therefore responsibility cannot be interventionist: ‘They make their own decisions, they’re the ones that take risks, and not to use condoms or do what they do and I can’t help them with that’ (Adam, 2005). This could be interpreted as a neoliberal feature of the well-informed sexual actor who minimises risks through the biomedicalisation of desire. Again, the overriding question that arises is: How can not wanting to contract HIV be constructed/interpreted as neoliberal? As happens in contemporary feminist and queer politics, the language of rights and choice—*my body, my choice*—aligns with neoliberal discourses of individualism and choice. These movements may use the language of individualism and choice, but it does not mean they are nothing more than neoliberal ideologies. It has more to do with how marginalised groups mobilise aspects of the dominant language to make their claims in a way that they can be heard. Therefore, the term *neoliberal* is not a helpful one when trying to understand gay men’s decisions regarding the use of antiretroviral medication for prevention or even their decisions to bareback.

In the same fashion that Race explained that the illicit drug user is a ‘useful figure for the neoliberal state’ because ‘it allows the state to individualise responsibility for those forms of consumption it deems to be immoral’ (Hakim and Race, 2023: 7), this paper argues that these types of sexual actors, often stigmatised, are not neoliberal, but neoliberalised in two dimensions. The first dimension is the application of the economics grid to understand sexual behaviour. The second is that, as is often the case in both the academic context and vernacular language, the term neoliberal is loaded with moral criticism, and this cannot be obviated. In other words, the term neoliberal is matched with concepts such as individualism, competition and self-investment, terms far removed from goodwill, solidarity, or investment in the community. Often, the term neoliberal becomes a moralistic label that positions its subject of study as a menace to public health and not as an opportunity to understand the complex decision process in which subjects make health-related decisions. Again, *wanting to be healthy or not sick* cannot be constructed/interpreted as neoliberal. Therefore, it is necessary to move beyond a pure neoliberal analysis to understand sexual practices in relation to HIV prevention.

At this point, there is a need to find a language that allows researchers to focus more on the messy aspects (racism, structural issues, marginalisation, inequities, ageism. . .) and less on the stigmatising and divisive modes of analysis based on neoliberal rhetoric. To put an example, the ‘risk calculator’ dimension attached to the neoliberal subjectivity is another adoption of an inherent activity of humans, which is calculating risk, that precedes neoliberalism and that tends to work as a stigmatising sineqdoque. As a variable, it is extremely limited to all the very intimate and complicated processes that affect decision-making. Bioconmensuration (Ecks, 2022) is a complex and less stigmatising model of analysis of decision-making processes. It has been defined as ‘processes that draw vitality, health, disease and healing into transactions with living and non-living entities’ (Ecks, 2022: 12), and its analysis includes questions regarding those entities that are transacted, or otherwise, and the recognition of multifactorial contextual dimensions that affect decision-making. What emerges from responding to those questions and acknowledging the complex contextualisation of sex scenarios is a more enlightened, more human figure than that of the neoliberal sexual actor. For example, taking PrEP, or not taking it is both complex biocommensurations. People who do not take PrEP or do not engage in safe sex use other criteria than public health mandates to make valuable comparisons. Bioconmensuration also decentralises personal responsibility as a moral determinant in sexual scenarios since it implies that decisions are taken to enhance life. As mentioned by Ecks (2021) ‘It is well known that neoliberalism shifts responsibility onto individuals, but how these supposedly responsibilized citizens are coping with the decision-making powers put upon them is much less explored’ (p. 408). Following this call to move beyond analysis focussing on neoliberal responsibilisation, I proceed to provide my biocommensuration process of pharmaceuticalised HIV governance.

## PrEP versus Sildenafil; a biocommensuration process

In December 2022, following two episodes of receptive condomless sex, I decided to schedule an appointment at the PrEP clinic in THT Brighton. While these two episodes served as a trigger to take antiretroviral drugs for prevention, I had, for years, deferred taking PrEP, engaging in a series of risk calculations to assess the likelihood of avoiding HIV acquisition. However, narrowing down the decision to take PrEP solely to these two episodes and my capacity to gauge risk and make informed choices fall short of capturing the intricacies of the valuation process. Since 2014, I have been aware of PrEP, and I have written both my MA and PhD dissertations on the subject of gay (bio)governmentality and PrEP. Through my research and interviews with PrEP activists and first-generation users, I fully understood the positive impact of PrEP on people’s lives and its contribution to the reduction of HIV incidence in England. Throughout these years, I consistently used condoms, thereby reducing the chances of acquiring the virus. Additionally, I was well-informed about the role of antiretroviral drugs in preventing HIV transmission through U = U.

However, I started to find it difficult to use condoms during casual sex due to reasons that included ‘failing biologies’ (Wahlberg, 2010), psychological and cultural issues, like Western ageism (Simpson, 2013), and my self-expectations to conform phallocratic masculinities. These factors can be linked to neoliberalism, much like racial capitalism, but they originate from patriarchal and phallocratic embodiments of sexuality, where penetrative sex is the hegemonic form. The pragmatic goal of my bio-commensuration process was to decide whether to use sildenafil for reliable erections to assist me in using condoms or to use PrEP, which would, in theory, release the pressure of having to use condoms. Both options would, to some degree, enhance my (sexual) life. In the calculations, I did not include the variable of being a receptive partner since this rarely occurred. However, when it did happen, it was the determinant factor to first be on PEP and then start PrEP. Unfortunately, I experienced constant and varying levels of nausea for 3 weeks as a side effect of taking daily PrEP, leading me to stop the regime. I decided to shift the protocol from daily PrEP to on-demand, but also to take sildenafil as necessary if I did not feel like taking PrEP. Finally, I chose to use only sildenafil, as I was able to resume using condoms consistently and efficiently. I did not weigh the economic factor in my decision, as PrEP is free at the point of delivery and I could afford the price of sildenafil (around £4per pill).

During the weeks that I took PrEP, I questioned myself, as I did with other participants in my study, about the role of responsibility while being on PrEP. It was clear that I was primarily taking PrEP for my own health benefit. This means, that I did not adopt a traditional public health approach in my PrEP taking, although I was aware that remaining negative (similar to remaining undetectable) contributed to the decreasing incidence of HIV. I agreed with those participants in my study who stated that protecting the community by taking PrEP is a side-effect of a decision based on individual needs. However, other types of questions related to hegemonic masculinities, intergenerational sex, self-esteem, and the need to perform acquired more relevance in terms of HIV prevention. For example, from a biomedical perspective, there has been an interest in researching the use of Sildenafil since the early 2000s due to its association with a higher risk of HIV infections, other sexually transmitted diseases, and even death when used with poppers. However, in my case, Sildenafil facilitated my condom use. The clinical dimensions of the biocomensuration process should be discussed with clinicians to improve health outcomes.

## Conclusion

This paper has examined the origins and the problematic effects that the concept of personal responsibility has had in the history of HIV prevention in the UK. As has been shown, the ubiquity of personal responsibility in HIV prevention debates in England has complicated its interpretation, since the concept has been used from very distanced, sometimes opposed, ideological perspectives. The concept becomes even more problematic when critiques of neoliberalism simplify the relationship between the concept of personal responsibility and collective action. In this sense, this criticism struggles to grasp the value of new forms of activism immersed in societies with markedly neoliberal health markets. For example, PrEP commodity activism uses formulae of the neoliberal system to access medicines, complicating the idea of activism as mere opposition to the neoliberal system (Martinez-Lacabe, 2021). PrEP commodity activism is a form of activism that links collective action with discourses on personal responsibility. However, far from being a paradox, it is the result of a multiplicity of human needs, desires and strategies to avoid barriers to medicine. In fact, the history of the concept of individual responsibility in isolation continues to produce stigmatisation of subjects of study when it is linked to representations of neoliberal subjectivities. As thoroughly illustrated in the article, the concept of the neoliberal sexual actor cannot work in real-life settings, as the complexity of sexual behaviour exceeds the few parameters aimed at characterising neoliberal ideology. The rationale of the neoliberal sexual actor is faulty and insufficient. This oversimplifying analysis deepens divisions between responsible and irresponsible gay men while obscuring the logic for participants engaging in unsafe sex. Contrary to the characterisation of this neoliberal subjectivity, its impact is not superfluous, as it has extended to other areas of health, including mothering and vaccination, resulting in equally stigmatised objects of study. Against this background, this article finally advocates moving beyond the polarised dichotomy of responsible/irresponsible subjects and further beyond the analysis of neoliberal subjectivities when addressing unsafe sex and evolving forms of understanding HIV prevention.
